# Recruiting Cancer Survivors to a Mobile Mindfulness Intervention in the United States: Exploring Online and Face-to-Face Recruitment Strategies

**DOI:** 10.3390/ijerph181910136

**Published:** 2021-09-27

**Authors:** Celine Isabelle Arnobit, Kiana Loo, Ian Pagano, Mai Uchiyama, Jami Fukui, Christa Braun-Inglis, Erin O’Carroll Bantum

**Affiliations:** 1 Cancer Prevention in the Pacific, University of Hawai‘i Cancer Center, Honolulu, HI 96813, USA; carnobit@hawaii.edu (C.I.A.); pagano@hawaii.edu (I.P.); maismith@hawaii.edu (M.U.); 2 School of Medicine, Case Western Reserve University, Cleveland, OH 44106, USA; ksl64@case.edu; 3 Cancer Biology, University of Hawai‘i Cancer Center, Honolulu, HI 96813, USA; JFukui@cc.hawaii.edu (J.F.); CBraunInglis@cc.hawaii.edu (C.B.-I.)

**Keywords:** recruitment, online, survivorship, mindfulness, mobile intervention, psychosocial

## Abstract

Cancer survivorship research faces several recruitment challenges, such as accrual of a representative sample, as well as participant retention. Our study explores patterns in recruited demographics, patient-reported outcomes (PROs), and retention rates for a randomized controlled trial (RCT) utilizing a mobile mindfulness intervention for the well-being of cancer survivors. In total, 123 participants were recruited using traditional and online strategies. Using the chi-square test of independence, recruitment type was compared with demographic and clinical variables, PROs, and retention at Time 2 and Time 3. Online recruitment resulted in almost double the yield compared to traditional recruitment. Online-recruited participants were more often younger, from the continental U.S., Caucasian, diagnosed and treated less recently, at a later stage of diagnosis, diagnosed with blood cancer, without high blood pressure, and with less reported pain. The recruitment method was not significantly associated with retention. Online recruitment may capture a larger, broader survivor sample, but, similar to traditional recruitment, may also lead to selection biases depending on where efforts are focused. Future research should assess the reasons underlying the higher yield and retention rates of online recruitment and should evaluate how to apply a mix of traditional and online recruitment strategies to efficiently accrue samples that are representative of the survivor population.

## 1. Introduction

### 1.1. Background

The population of cancer survivors is rapidly increasing. There were more than 16.9 million Americans with a history of cancer as of January 1, 2019, and this number is expected to increase to 22.1 million by 2030 [1]. Research to promote quality of life and well-being in the growing cancer survivor population will only become more essential and relevant.

Despite its importance as an increasingly relevant field of study, cancer survivorship research in general is difficult to recruit for [2,3,4], regardless of the intervention format.

It is especially difficult to recruit a representative sample that reflects the clinical and demographic diversity of the survivor population. Many subgroups are historically underrepresented in psychosocial oncology, signaling selection bias, such as survivors from rural areas [3], adolescent and young adult (AYA) survivors [5,6], and racial and ethnic minority survivors [7].

Recruitment is an essential component of the research process, and its success has direct impacts on the study’s sample size, bias, and power [2,5,8,9]. In general, however, studies in the field of psychosocial oncology sparsely report recruitment data [4], necessitating further literature which critically examines participant accrual and its subsequent effects on the study, such as regarding retention.

A variety of barriers may impede study participation and retention in survivorship interventions despite a vast population of potentially eligible participants. For participants, systematic barriers include financial challenges/constraints, deteriorating or poor health conditions, physical limitations, and geographic barriers (i.e., need to travel to a research facility) [2,4,10]. There may also be barriers related to patient attitudes, such as a preference for specific treatments, perceived patient burden of participation in the study, or low actual desire for treatment despite reporting psychological distress [4,10,11].

### 1.2. Strengths and Limitations of Online Recruitment Methods

Online recruitment methods may overcome several of these barriers and provide easier access to participation. Internet-based recruitment methods—such as email, websites, and social media—have some advantages and are becoming increasingly popular in health research [12,13]. These contrast with traditional or offline recruitment methods, such as physical flyer distribution, mailings, traditional media coverage including radio or TV, and clinic-based or registry-based recruitment [3,5].

Online recruitment strategies can be more efficient and effective than traditional methods in terms of participant enrollment numbers [14,15]. In addition, they are more resource and time-efficient [2,7,15], and allow for recruitment of participants from a broader geographic range, such as from remote and rural places [9,15,16]. Other studies also find that online recruitment for health research—in particular, through social media—expands the breadth of reach for recruitment and is able to recruit “hard-to-reach” populations [7,15], particularly those with specific conditions or disorders [15], such as cancer survivors. Within the cancer survivor population, online strategies have also been shown to recruit participants with a larger range of clinical conditions, such as more variable cancer diagnoses and treatment statuses [2,5].

Despite their ability to expand the breadth of reach, online recruitment methods also come with inherent selection biases that merit further exploration. For example, online recruitment in psychosocial interventions resulted in the accrual of samples that were skewed towards younger [2,17,18], female [2,17,18,19], White [5,17,19], and higher-educated [5,17,18] demographics, as well as breast cancer survivors [2,5,17,19]. Regarding baseline characteristics, participants recruited online for the studies of Owen et al. [18] and Benedict et al. [5] were also found to report poorer psychosocial wellbeing on variables of interest than that of traditionally recruited participants.

### 1.3. The Current Study

This study aims to further explore such biases in demographics and patient-reported psychological outcomes, and to evaluate whether these biases exist within our own study, which is an online mindfulness intervention for the well-being of cancer survivors. In addition, we aim to evaluate study retention rates in relation to the recruitment strategy.

To achieve this, we conducted a preliminary analysis of recruitment data from a randomized controlled trial (RCT) called the “Mobile Mindfulness Intervention to Promote the Well-being of Cancer Survivors.” This trial began in April 2019 and is still conducting active recruitment. The data collection for the current analysis is as of February 2021. 

In the parent study, participants were provided a mindfulness intervention via a mobile app to improve anxiety and other markers of well-being in cancer survivors. The objective of the current analysis is to evaluate the efficacy of the online recruitment methods used for the study, which is measured by recruitment numbers and retention rates, as well as to examine any patterns in the recruited participant demographics. 

The rarity of online methods in recruitment for mental health studies [9], coupled with the challenges of recruiting a cancer survivor population [2,3,4] and a relative paucity of health science literature that reports recruitment data [4,8], has resulted in few studies that have been able to analyze online recruitment and retention for an online psychosocial intervention. Our study seeks to add to the existing research by posing the following questions:

1. Does the recruitment method impact retention rates?
2. Does the recruitment method influence sample demographics and characteristics?
3. Which recruitment methods prove most effective in recruiting and retaining participants?

We hypothesize that: (1) online recruitment methods, specifically social media, will yield higher participant enrollment but lower retention rates; (2) traditional recruitment methods will yield lower participant enrollment but higher retention rates; and (3) we will detect significant differences in patient baseline characteristics by recruitment type.

## 2. Materials and Methods

### 2.1. Mobile Mindfulness Intervention to Promote the Well-Being of Cancer Survivors

The parent study is an RCT utilizing a mindfulness meditation mobile app for cancer survivors with anxiety. The mobile app used in the parent intervention is called Mindfulness Coach. It was developed by the VA National Center for PTSD in collaboration with the National Center for Telehealth and Technology (T2), and with the participation of Dr. Erin Bantum. Mindfulness Coach includes a brief tutorial, a training plan, practice exercises, learning topics, and a tracking function. There are 14 sessions, each culminating in a meditative practice. This study is being conducted at the University of Hawai‘i Cancer Center. 

The study assesses patient-reported outcomes (pain, fatigue, anxiety, depression, trauma, and mindfulness) through online questionnaires at baseline (Time 1), with follow-up surveys at eight (Time 2) and sixteen weeks (Time 3). The study has a wait-list control design, with the treatment group receiving access to use the app at baseline and the wait-list group receiving access to the app at eight weeks. The study has been approved by the University of Hawai‘i Internal Review Board (IRB).

Although active recruitment for the intervention is ongoing at the time this paper is written, for the purposes of the current study, recruitment data from April 2019 to February 2021 will be analyzed.

### 2.2. Participants

During the initial survey, participants are screened for eligibility criteria. Participation in the study requires that participants must be: cancer survivors (excluding non-melanoma skin cancer) that have completed primary treatment (hormone therapy accepted); over 21 years of age; experiencing at least a mild level of anxiety at the time of enrollment, as measured by a minimum score of 22 on the Patient-Reported Outcomes Measurement Information System (PROMIS) scale [20]; have access to a mobile device; comfortable reading and writing in English; and not currently practicing more than one hour of meditation a day.

### 2.3. Measures

#### 2.3.1. Participant Demographics

After completing the screening survey and consenting to study participation, participants complete a demographic survey, which asks information related to sex, gender identity, age, birthplace, where they currently live (Hawai‘i, continental U.S., or “other”), ethnic background, education level, employment status, and marital status.

The survey also asks about clinical characteristics such as diagnosis date, treatment completion date, cancer type, cancer stage, type of treatment(s) received, and additional medical conditions.

#### 2.3.2. Recruitment Method

We evaluated recruitment methods using a question embedded in the demographics survey: “How did you hear about this study?” The responses were in the free-response format. Participant responses were categorizable into one of six recruitment strategies:

Traditional recruitment methods: (1) clinical/hospital recruitment, (2) support group, and (3) friend/family.

Web-based recruitment methods: (4) email, (5) social media, and (6) miscellaneous online method (if participant did not specify details about web-based recruitment; also includes online support groups).

#### 2.3.3. Baseline PROs

The Time 1 survey measures several patient-reported outcomes (PROs): pain, measured with the PEG-3 [21]; fatigue, measured with the Brief Fatigue Inventory (BFI-9) [22]; anxiety, measured with the General Anxiety Disorder scale (GAD-7) [23]; depression, measured with the Patient Health Questionnaire (PHQ-9) [24]; trauma, measured with the Posttraumatic Stress Disorder Checklist (PCL-5) [25]; and mindfulness, measured with the Five Facet Mindfulness Questionnaire (FFMQ-39) [26].

#### 2.3.4. Retention

Participant retention was measured as the percentage of participants who completed their follow-up surveys at Time 2 and at Time 3.

### 2.4. Recruitment Strategies

A study flyer was created containing basic information, including eligibility criteria, link and QR code to the online survey, and contact information for the research team. It received IRB approval and was distributed both locally (statewide) and nationally through various methods. Recruitment strategies can be categorized into two groups: traditional recruitment methods and web-based recruitment methods.

#### 2.4.1. Traditional Recruitment Methods

*Clinical and hospital-based recruitment:* The study flyers were printed and given to several outpatient clinics and healthcare providers in the state of Hawai‘i. The majority of the clinicians and support groups that helped with recruitment have had contact or affiliation with the University of Hawai‘i Cancer Center prior to this study. Some of the recruitment methods within this category included a presentation at virtual support groups and direct patient referral to the study by health care providers during oncology follow-up appointments.

*University of Hawai‘i Cancer Center:* The study was also promoted at an internal seminar hosted by the University of Hawai‘i Cancer Center faculty, as well as during internal committee meetings. In addition, the study has been posted on the University of Hawai‘i Cancer Center website and presented at multiple meetings (e.g., a regional Leukemia and Lymphoma Society meeting).

#### 2.4.2. Web-Based Recruitment Methods

A total of 90 groups—including cancer support groups, health centers, and non-profit organizations—were contacted via web-based recruitment methods during the recruitment period. Web-based recruitment methods can be further categorized into email and social media methods.

*Email*: Templated emails providing study information and accompanying study flyers were used to initiate contact with organizations. Most organizations that were contacted then disseminated the study flyer and relevant recruitment language through formats such as a listserv, closed email listing, e-newsletter, or post on private online forums.

*Social media:* Some organizations, after initial contact and correspondence via email, disseminated the study information to their members via their public social media accounts. These organizations also received the flyer and approved the recruitment language to post to their social media accounts, which primarily were Instagram and/or Facebook.

Social media was also used as a means of primary recruitment. Members of the research team directly posted the recruitment language for the study on the public Facebook pages of different cancer non-profit organizations and other organizations under their prospective “Community” tabs, which is a subsection on Facebook that allows users who visit the organization’s page to publicly post messages on the organization’s “wall.” The research team also directly messaged the administrators of various cancer group Facebook pages as a means of initiating contact with the organization. With the exception of two organizations, however, the majority of organizations did not engage with the “Community” page postings nor respond to the direct messages.

### 2.5. Statistical Analysis

We compared recruitment type (traditional vs. online) with demographic and clinical variables, as well as PROs at Time 1 using the chi-square test of independence. Demographic variables were sex, age, place of residence (Hawai‘i, continental U.S., or other), ethnicity, education level, employment status, and marital status. Clinical variables were years since diagnosis, years since treatment, cancer type, stage, type of treatment received, and other medical conditions. PROs examined were pain (PEG-3), fatigue (BFI-9), anxiety (GAD-7), depression (PHQ-9), trauma (PCL-5), and mindfulness (FFMQ-39). Retention rates at Time 2 and Time 3 were compared with recruitment type and group assignment for significant associations.

The chi-square test of independence determined statistically significant (*p* < 0.05) differences. PRO variables were categorized into quartiles. The SAS 9.4 software (SAS Institute Inc., Cary, NC, USA) performed all data management activities and analyses.

## 3. Results

In total, 123 participants were recruited and completed the baseline survey between 1 April 2019 and 18 February 2021, which was the cutoff date for this analysis.

For recruitment analyses, through the recruitment question in the survey, we ascertained that 77 participants were recruited online and 43 through traditional strategies. Recruitment method data was missing for three participants who either did not answer or were from an indiscernible recruitment source. These were marked as “missing data” and were excluded from the analyses, leaving a final sample total of *N* = 120 that were analyzed by recruitment method (see Table 1).

For retention analyses, retention data (i.e., whether or not a participant completed their follow-up surveys) was missing for one participant at Time 2 and for six participants at Time 3 due to participants not yet having been due for their respective follow-up surveys at the time of the statistical analyses. This led to a total of 122 participants who had retention data for Time 2 and 117 participants who had retention data for Time 3.

### 3.1. Participant Demographics and PROs

Our sample was mostly female (83.3%), Caucasian (60.0%), over the age of 35 (79.2%), lived in the continental U.S. at the time of survey (53.3%), and had a college degree or higher (74.2%).

Nine variables (age, residence, ethnicity, years since diagnosis, years since treatment, cancer type, cancer stage, high blood pressure, and the PEG-3) showed statistically significant differences (*p* < 0.05) between the traditional and online recruitment methods (see Table 1 and Table 2.) Compared with participants who were recruited using traditional methods, online recruits were more often younger (*p* = 0.005), from the continental U.S. (*p* = 0.009), Caucasian (*p* = 0.001), diagnosed and treated less recently (*p* = 0.02 and *p* = 0.01, respectively), at a later stage of diagnosis (*p* = 0.04), without high blood pressure (*p* = 0.04), and with lower PEG-3 scores (*p* = 0.02). They were less often diagnosed with breast, colorectal, or lung cancer, and more often diagnosed with blood cancer (*p* = 0.0009).

### 3.2. Retention

**Figure 1:**
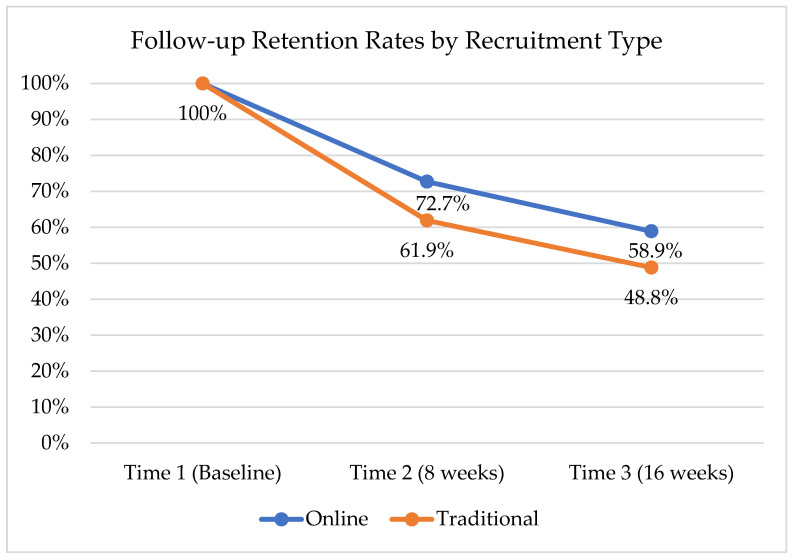
Overall retention by recruitment type.

Recruitment method was not found to have an impact on participant retention rates at Time 2 and Time 3. However, generally, retention rates were higher for online-recruited participants: at Time 2, traditional recruitment methods yielded 61.9% retention and online methods yielded 72.7% retention. At Time 3, traditional recruitment methods yielded 48.8% retention, while online methods yielded 58.9% retention (see Table 1 and Figure 1).

Group assignment was associated with the completion of the follow-up survey at Time 2 but not during Time 3, such that controls were more likely than the treatment group to complete the Time 2 survey (*p* = 0.03). Retention at Time 2 was associated with retention at Time 3 (*p* < 0.0001), such that those who completed the Time 2 survey were also more likely to complete the Time 3 survey (see Table 3).

## 4. Discussion

### 4.1. Participant Demographics and PROs

In psychosocial oncology specifically, online recruitment methods may be more effective than traditional recruitment methods at representing a broader range of the cancer survivor population, as well as more diversity in clinical characteristics, although there is some nuance to be noted. Our online-recruited group consisted of a larger range of diagnoses compared to the traditionally recruited group. The most common cancer diagnoses in participants recruited online were “Other” (i.e., not blood, breast, colorectal, or lung cancer; 48.1%), compared with the traditionally recruited group, whose most common cancer type was breast cancer (51.2%). Hulbert-Williams et al. similarly found that when conducting recruitment for an Acceptance and Commitment Therapy intervention for cancer survivors, social media and other online methods resulted in a more heterogeneous sample than is normally seen in psychosocial oncology and included a wider range of both cancer diagnoses and treatment statuses [2]. Benedict et al. also noted in their study that online recruitment for an AYA fertility study led to a sample with more diverse cancer diagnoses than that recruited through hospitals [5]. Considering online interventions, such as Mindfulness Coach, have the potential to positively impact cancer survivors, adequate recruitment to ensure that participants garner that benefit is important.

However, it is crucial to note that our highest-accruing method of traditional recruitment was in-clinic recruitment (39.5% of traditional recruits were recruited in-clinic), mostly through breast cancer healthcare providers, which could have resulted in a breast cancer bias for traditional recruitment. Indeed, only about a third of all online-recruited participants were breast cancer survivors, while more than half of all traditionally recruited participants were breast cancer survivors.

Furthermore, online recruitment more frequently recruited blood cancer survivors than the traditional methods (*p* = 0.0009). This can be attributed to the fact that we recruited a large portion of our participants online through social media with a well-known AYA cancer non-profit organization and considering the fact that leukemia is among the most common cancers in the AYA population [27].

Our online-recruited group reflected some demographic patterns seen in past literature: as in other studies, our online group tended to be younger [2,15,17,18], Caucasian/White [5,17,19], had received treatment less recently [5], and were diagnosed less recently [18]. Although significant differences were not found in sex and education level, our online methods generally trended towards more often recruiting females and the higher educated (*p* = 0.15 and *p* = 0.09, respectively) than the traditional methods, which is congruent with patterns seen in other psychosocial oncology recruitment studies [2,5,15,17,18,19].

Although our findings were reflective of those from several studies, it should be noted that they are also contradictory to others. Keaver et al. found, for example, that their online-recruited sample tended to be diagnosed more recently (i.e., within 5 years of initial diagnosis, as opposed to survivors diagnosed >5 years ago) [17]. Another pattern that is frequently seen in online-recruited cancer survivor samples is a breast cancer bias [2,5,17,19], which we did not see in our own online-recruited group, which more commonly recruited participants with “Other” cancer types (48.1%).

Our online-recruited group differed on variables of interest only in that they reported less pain as indicated by lower PEG-3 scores, possibly because this group also tended to be further from treatment. No significant differences were found on other psychosocial measures (anxiety, depression, trauma, fatigue, and mindfulness). Our results contrast with other studies which found that participants recruited online reported increased distress and worse psychosocial well-being [5,18]. This discrepancy merits further study to determine if a consistent pattern persists regarding recruitment method and psychosocial measures of well-being. It is important to contextualize these findings within the United States. While social media and recruitment through social networking, as well as through interventions that are commonly offered such as mindfulness meditation, can be generalized to a certain extent, there are likely some differences that would be found internationally.

### 4.2. Retention

The type of recruitment method, either traditional or online, did not have a significant association with participant retention rates, suggesting that recruitment source may not be necessarily related to participant retention. We did, however, find an association between group assignment and retention, such that our control group had higher retention rates for Time 2. In survivorship research, there may be several factors that affect study retention other than disease progression or death, including perception of patient burden [28,29] and preference for group assignment [29,30]. In our study, we do not collect data about such variables related to participant attitudes and thus are unable to determine individual reasons for attrition, a facet of study recruitment that merits further exploration.

We also observed an association between the completion of the Time 2 survey and the completion of the Time 3 survey, signaling that participants who are successfully retained until Time 2 are also likely to continue for the duration of the study. It thus may be crucial for researchers conducting studies with multiple follow-ups to implement strategies that promote high retention for the first follow-up survey, as it may impact the rates of retention for subsequent follow-ups.

### 4.3. Strengths and Limitations

The overall design of the study allowed for a fair comparison of participation demographics and outcomes based on recruitment type. The ability to analyze both eight and sixteen-week survey points allowed for two separate time points to quantify the impact of recruitment type on retention, as well as allowed for the assessment of other factors related to retention, such as the group. Our study is also one of the relatively few to critically report and evaluate the efficacy of online recruitment methods specifically for an online psychosocial intervention.

Another strength of our study is that we had a substantial amount of participants (42.5% of the total sample) who currently live in Hawai‘i. This is significant because the general population of the state is generally more racially and ethnically diverse compared to that of the continental U.S. [31]. Thus, studies done mostly with samples from the continental U.S. cannot necessarily be generalized to Hawai‘i’s population, whose demographic diversity merits exploration in its own right.

Some limitations of our study include a relatively small sample size, the categorization of online and traditional recruitment methods, and the potential impact of the COVID-19 pandemic. It is also important to note that by its nature, the sample is somewhat biased in the sense that we are recruiting people who are interested in these interventions. There are likely differences between this group and the population of cancer survivors at large.

A larger sample size would be ideal for better examining demographic trends and generalizing results to greater national and international cancer survivor populations. It would also be worth analyzing a sample with a specific focus on Hawai‘i’s own demographics, such as a sample whose majority is comprised of local residents, to explore how and if patterns in large-scale psychosocial oncology research are also applicable to Hawai‘i residents.

Additionally, the formatting of the “How did you hear about this study?” as a free-response question required the research team to discern recruitment methods of participants through cross-referencing and double-checking participant responses against our own records of modes of recruitment (which was done successfully, apart from the three participants excluded from our analyses). A multiple-choice option for this survey item would have expedited the process of coding recruitment sources, as well as lowered the chances of potentially mis-categorizing participant responses.

#### 4.3.1. Impact of COVID-19

Implementing this study during the COVID-19 pandemic could have also influenced the outcomes, as 76 of the 123 participants were recruited during the COVID-19 pandemic (March 2020 onwards), with 51 of these recruited through online methods. Prior to the pandemic, between April 2019 and February 2020, 44 participants had been recruited, of which 26 were through online recruitment. Due to the limited in-person interaction during the COVID-19 pandemic, the options to recruit traditionally during this time were sparse. The majority of recruitment efforts were focused primarily on online recruitment for this reason, although in-clinic recruitment by healthcare providers occurred where possible during this time. Thus, the logistical challenges of traditional recruitment during the COVID-19 pandemic could be a possible reason for a greater number of online recruitments than traditional recruitments in this analysis.

Additionally, changes in the availability and potential stress of participants could have also influenced the rates of recruitment and retention. The pandemic could have contributed to higher rates of anxiety, making our study more attractive for potential participants. Moreover, since much of the recruitment during the pandemic was online, joining the study at this time may have been more opportune for participants since they are able to participate remotely during a time when all were encouraged to stay home.

As a result of the pandemic, rates of telemedicine have accelerated and are suggested to leave lasting impacts on the advancement and norms of healthcare [32,33]. This shift to more Internet-based healthcare could potentially impact the future of online health interventions. With continued decreased rates of in-person health visits and increased rates of telemedicine [34,35], web-based psychosocial interventions, such as Mindfulness Coach, may be more widely used and perhaps may become more attractive alternatives to in-person treatment for those who choose to continue utilizing telehealth for the foreseeable future. Consequently, if lower rates of in-person health visits continue, online methods of recruitment may become more applicable for researchers than traditional hospital and clinic-based strategies.

#### 4.3.2. Fraudulent Users

Additionally, given the impersonal nature of online recruitment for web interventions, there remains the challenge of the potential for fraudulent users to enroll in the study dishonestly [8,9,15], especially if there is an incentive to participate.

In our study, we had two relatively large waves of fraudulent users during late July and early November 2020 of roughly about 170 users in total. Based on recruitment activity during these periods, we believe these users may have been attracted as a result of a social media posting by a cancer organization with a large following that we had contacted for recruitment support. The post included mention of the monetary study incentive, which may have tempted fraudulent users to enroll.

Potential fraudulent users were identified by repetitive IP addresses and incongruent demographic and questionnaire answers (for example, disclosing that they were a Vietnam war veteran when their date of birth showed that they would not have been alive at the time). Potential fraudulent users were then contacted via email by the research team to confirm their authenticity in case that they were miscategorized. All confirmed fraudulent users were subsequently removed from the study and dataset. Following the influx of fraudulent users, we altered our recruitment language with IRB approval so as not to publicly advertise the study incentive on social media postings.

Although it remains unclear how to completely eliminate the risk of fraudulent users in online interventions, it may be prevented by avoiding advertising monetary incentives on social media and other public Internet recruitment messages. In addition, keeping accurate track of recruitment contacts and dates can help researchers identify potential online sources of fraudulent users should they occur, as well as help to respond quickly and accordingly to protect the study integrity. As online interventions become more commonplace, it may be advantageous for studies to report rates of fraudulent participants as well as factors involved in successful and honest Internet-based recruitment.

### 4.4. Suggestions and Future Directions for Online Recruitment

Much of our success during recruitment was dependent on the extent of engagement that organizations had with their followers, as well as the relationship between the organization and the recruitment team. Support groups, for example, seemed to be more engaged with recruitment and its members were more receptive to spreading the word about our study with one another, perhaps due to the rapport and trust among members. Recruitment through one’s healthcare provider may also be more motivating for enrollment and consistent follow-up due to the familiarity of the provider–patient relationship. Indeed, in our study, locally based groups and clinic-based contacts were more engaged and responsive to both recruitment types, perhaps due to the familiarity with the University of Hawai‘i Cancer Center as a local institution. Future studies could continue to examine the relationships between recruiter, liaisons, and study participants to identify tenets of successful recruitment.

Additionally, although notable for its low financial cost and high participant yield as a recruitment tool [14], social media remains a relatively uncommon recruitment strategy in psychosocial oncology [2]. This may be because the cancer survivor population is generally older and thus may be less apt to use social media; however, because this trend is changing as technology becomes increasingly pervasive among all populations, social media’s potential as a recruitment tool should be further explored.

Future research should also assess reasons underlying the higher yield and retention rates of online recruitment in general, as well as should further examine the demographic trends and biases that may arise through online strategies.

Although no pattern was observed here, the relationship between recruitment and retention should also be explored and we are curious if this continues to be the case in other psychosocial interventions. There is sparse literature that reports on the retention rates for health studies that utilize at least one method of online recruitment [8,30,36] and even fewer specifically for survivorship studies.

We suggest that future online health research, especially within psychosocial oncology, report successful recruitment tactics, demographics of recruited samples, and retention rates, which can serve as a proxy measure of recruitment success [8]. These are crucial data in evaluating the extent of intervention delivery among various demographics within the survivor population, as well as in evaluating where there are inadvertent opportunities for selection bias to influence intervention efficacy. Finally, the reporting of such data can inform the future development of recruitment strategies that are efficient and efficacious for online interventions, allowing for mHealth survivorship interventions to benefit as many people as possible.

## 5. Conclusions

Online recruitment methods can be useful tools when used in conjunction with face-to-face strategies to address barriers to research participation within the cancer survivor population. The nature of online recruitment lends itself well to widening the breadth of potential study participants and making online interventions, such as our mHealth app for mindfulness meditation, more accessible to a greater range of cancer survivors. Indeed, findings from our study support previous literature which find that online recruitment results in a more diverse range of cancer diagnoses. However, some recruitment strategies also potentially give rise to inadvertent selection biases related to participant demographics, depending on where recruitment efforts are focused, which can affect study outcomes. Our study has aimed to fill the paucity of research surrounding online psychosocial oncology recruitment, an area which remains critical to address in an increasingly technologically dependent world, where the needs and well-being of cancer survivors must not be overlooked.

## Figures and Tables

**Table 1:** Participant demographics and clinical characteristics by recruitment type.

		Traditional		Online				
		(*N* = 43)		(*N* = 77)				
Variable	Value	*N*	%	*N*	%	χ^2^	df	*p*
Group	Control	21	48.8	37	48.1	0.0	1	0.93
	Mindfulness App	22	51.2	40	51.9			
Recruitment source								
	Clinical recruitment	17	39.5					
	Traditional support group	12	27.9					
	Friend/family	14	32.6					
	Email			25	32.5			
	Social media			47	61.0			
	Other online method			5	6.5			
Retention								
Completed T2 Survey	No	16	38.1	21	27.3	1.5	1	0.22
	Yes	26	61.9	56	72.7			
Completed T3 Survey	No	21	51.2	30	41.1	1.1	1	0.30
	Yes	20	48.8	43	58.9			
Participant Demographics								
Sex	Female	33	76.7	67	87.0	2.1	1	0.15
	Male	10	23.3	10	13.0			
Age	18–34	2	4.7	23	29.9	10.6	2	**0.005**
	35–54	22	51.2	29	37.7			
	55+	19	44.2	25	32.5			
Currently Live	Hawai‘i	26	60.5	25	32.5	9.4	2	**0.009**
	Continental U.S.	15	34.9	49	63.6			
	Other	2	4.7	3	3.9			
Ethnicity	Asian	12	27.9	10	13.0	16.1	3	**0.001**
	Caucasian	16	37.2	56	72.7			
	Hawaiian/part-Hawaiian	6	14.0	2	2.6			
	Other	9	20.9	9	11.7			
Education	No college degree	15	34.9	16	20.8	4.7	2	0.09
	College degree	20	46.5	34	44.2			
	Graduate degree	8	18.6	27	35.1			
Employment	Full-time	18	41.9	36	46.8	0.3	3	0.95
	Part-time	6	14.0	11	14.3			
	Retired	8	18.6	13	16.9			
	Unemployed	11	25.6	17	22.1			
Marital Status	Living with a partner	5	11.6	12	15.6	4.5	3	0.21
	Married	25	58.1	40	51.9			
	Past partner	8	18.6	7	9.1			
	Single, never married	5	11.6	18	23.4			
Years Since Diagnosis	0–2	12	29.3	18	23.4	8.4	2	**0.02**
	2–5	23	56.1	28	36.4			
	5+	6	14.6	31	40.3			
Years Since Treatment	0–2	27	65.9	32	42.7	8.8	2	**0.01**
	2–5	11	26.8	21	28.0			
	5+	3	7.3	22	29.3			
Cancer Type	Blood	1	2.3	10	13.0	18.6	4	**0.0009**
	Breast	22	51.2	25	32.5			
	Colorectal	4	9.3	5	6.5			
	Lung	5	11.6	0	0.0			
	Other	11	25.6	37	48.1			
Stage	0–1	22	53.7	18	26.5	8.3	3	**0.04**
	2	9	22.0	22	32.4			
	3	8	19.5	21	30.9			
	4	2	4.9	7	10.3			
Type of Treatment Received								
Surgery	No	9	20.9	13	16.9	0.3	1	0.58
	Yes	34	79.1	64	83.1			
Chemotherapy	No	8	18.6	18	23.4	0.4	1	0.54
	Yes	35	81.4	59	76.6			
Radiation	No	21	48.8	41	53.2	0.2	1	0.64
	Yes	22	51.2	36	46.8			
Hormonal	No	26	60.5	53	68.8	0.9	1	0.35
	Yes	17	39.5	24	31.2			
Other Medical Conditions								
Arthritis	No	29	80.6	49	89.1	1.3	1	0.26
	Yes	7	19.4	6	10.9			
Asthma	No	26	72.2	40	72.7	0.0	1	0.96
	Yes	10	27.8	15	27.3			
Back Problems	No	26	72.2	42	76.4	0.2	1	0.66
	Yes	10	27.8	13	23.6			
Diabetes	No	32	88.9	50	90.9	0.1	1	0.75
	Yes	4	11.1	5	9.1			
Emphysema	No	36	100.0	54	98.2	0.7	1	0.42
	Yes	0	0.0	1	1.8			
Heart Disease	No	34	94.4	54	98.2	1.0	1	0.33
	Yes	2	5.6	1	1.8			
High Blood Pressure	No	20	55.6	42	76.4	4.3	1	**0.04**
	Yes	16	44.4	13	23.6			
Other Problems	No	21	58.3	33	60.0	0.0	1	0.87
	Yes	15	41.7	22	40.0			

Statistically significant differences (*p* < 0.05) indicated in bold.

**Table 2:** Comparing PROs by recruitment type.

	Traditional					Online					
PRO	*N*	Mean	SD	Min	Max	*N*	Mean	SD	Min	Max	*p*
PHQ-9	43	19.7	4.8	10	30	77	18.8	5.1	9	34	0.32
GAD-7	43	16.7	3.9	11	28	77	16.7	4.1	8	28	0.93
PCL-5	43	44.2	12.3	26	73	77	41.8	13.1	23	82	0.32
PEG-3	43	12.6	7.2	0	30	76	9.3	7.8	0	30	**0.02**
BFI-9	43	44.5	19.5	6	86	76	38.1	20.2	1	84	0.09
FFMQ-O 8 ^1^	43	26.3	5.0	13	37	77	25.3	5.3	13	38	0.33
FFMQ-D 8 ^2^	43	26.7	6.2	16	40	77	27.4	6.3	15	40	0.57
FFMQ-A 8 ^3^	43	24.5	4.0	14	35	77	24.2	5.1	15	40	0.77
FFMQ-NJ 8 ^4^	43	24.8	7.8	14	40	77	26.1	6.2	10	39	0.37
FFMQ-NR 7 ^5^	43	21.0	3.4	13	28	77	20.2	3.6	10	29	0.19
FFMQ-39 ^6^	43	123.3	15.2	101	168	77	123.1	17.0	84	166	0.96

Statistically significant differences (*p* < 0.05) indicated in bold. ^1^ “Observing” subscale (eight items); ^2^ “Describing” subscale (eight items); ^3^ “Acting with Awareness” subscale (eight items); ^4^ “Non-judging of Inner Experience” subscale (eight items); ^5^ “Non-reactivity to Inner Experience” subscale (seven items); and ^6^ overall FFMQ-39 score.

**Table 3:** Comparing retention rates by recruitment type (one participant missing data for Time 2; six participants missing data for Time 3).

Variable	Value	Statistic	Total	No	Yes	χ^2^	df	*p*
Completed T2 Follow-up								
	Total	*N* (%)	122	37 (30.3)	85 (69.7)			
Group	Control	*N* (%)	58	12 (20.7)	46 (79.3)	4.9	1	**0.03**
	Mindfulness App	*N* (%)	64	25 (39.1)	39 (60.9)			
Recruitment Method	Traditional	*N* (%)	42	16 (38.1)	26 (61.9)	1.5	1	0.22
	Online	*N* (%)	77	21 (27.3)	56 (72.7)			
	Missing data	*N* (%)	3	0 (0.00)	3 (7.1)			
Completed T3 Follow-up								
	Total	*N* (%)	117	52 (44.4)	65 (55.6)			
Group	Control	*N* (%)	54	20 (37.0)	34 (63.0)	2.2	1	0.14
	Mindfulness App	*N* (%)	63	32 (50.8)	31 (49.2)			
Recruitment Method	Traditional	*N* (%)	41	21 (51.2)	20 (48.8)	1.1	1	0.30
	Online	*N* (%)	73	30 (41.1)	43 (58.9)			
	Missing data	*N* (%)	3	1 (0.9)	2 (1.7)			
Completed T2 Follow-Up	No	*N* (%)	37	37 (100.0)	0 (0.00)	67.6	1	**<0.0001**
	Yes	*N* (%)	80	15 (18.8)	65 (81.3)			

Statistically significant differences (*p* < 0.05) indicated in bold.

## Data Availability

Any requests for data should be directed to the corresponding author due to participant confidentiality. Data has been stored securely by the University of Hawai‘i Cancer Center.
